# Preschool Communication: Early Identification of Concerns About Preschool Language Development and Social Participation

**DOI:** 10.3389/fpubh.2020.546536

**Published:** 2021-01-22

**Authors:** Bernice M. Doove, Frans J. M. Feron, Jim van Os, Marjan Drukker

**Affiliations:** ^1^Youth Health Care Division, Regional Public Health Service South Limburg, Heerlen, Netherlands; ^2^Department of Social Medicine, Care and Public Health Research Institute, School for Public Health and Primary Care, Maastricht University, Maastricht, Netherlands; ^3^King's Health Partners, Department of Psychosis Studies, Institute of Psychiatry, King's College London, London, United Kingdom; ^4^Department of Psychiatry and Psychology, MHeNS School for Mental Health and NeuroScience, Maastricht University Medical Centre, Maastricht, Netherlands; ^5^Department Psychiatry, Brain Center Rudolf Magnus, Utrecht University Medical Centre, Utrecht, Netherlands

**Keywords:** preschool social participation, social competence, communication, early identification, language concerns, PEDS, personalized health care, monitoring

## Abstract

**Background:** Adverse communication development in preschool children is a risk factor influencing child health and well-being with a negative impact on social participation. Language and social skills develop and maintain human adaptability over the life course. However, the accuracy of detecting language problems in asymptomatic children in primary care needs to be improved. Therefore, it is important to identify concerns about language development as a risk factor for child health. The association between parental and professional caregivers' concerns about language development and the level of preschool social participation was assessed, as well as the possible mediating/moderating effect of the perception of social competence. In addition, validity and predictive value of parental and professional caregivers' concerns about language development were tested.

**Methods:** To identify emerging concerns about development and social participation, a community sample of 341 preschool children was systematically assessed with a comprehensive preventive child health care “toolkit” of instruments, including parent-completed tools like the Parents' Evaluation of Developmental Status (PEDS) and child competence Visual Analog Scales (VAS). At baseline, children were aged 3 years and at follow-up ~4 years.

**Results:** There was a statistically significant association between parental and professional caregivers' concerns about language development and the level of preschool social participation, with a mediating effect of child social competence at the age of 3 years as well as 4 years. Negative predictive value of parental and professional caregiver language concerns at the age of 3 and 4 years were 99 and 97%, respectively. Furthermore, this article showed that while some preschool children grow out of language problems, others may develop them.

**Conclusion:** Short but valid pediatric primary care tools like the PEDS and child competence VAS can support monitoring and early identification of concerns about language development and social competence as a risk factor for preschool social participation. Personalized health care requires continued communication between parents, professional caregivers and preventive child health care about parental and professional caregiver perceptions concerning preschool language development as well as the perception of a child's social competence.

## Introduction

Poor communication is a risk factor influencing child health and well-being with adverse consequences for behavior, literacy, learning, mental health, future employment, parenting, the next generation, and social inequalities (1, 2).

Effective communication is fundamental to the initiation and maintenance of successful peer relations (3, 4). The ability to interact with others and to establish relationships is of great influence on learning and development, and successful social adaptation and participation. From a dynamic perspective, health can be seen as the ability to adapt and self-manage in the face of social, physical, and emotional challenges (5). For this, language and social skills are needed; they develop and maintain human adaptability over the life course (6, 7).

From a public health perspective, preschool children represent an important group (8, 9). The preschool period is a sensitive period in language development (7, 10). Developmental growth in language skills is an important parameter of overall communication development (11). Language problems are often the first presenting symptoms of delay in the development of multiple basic functions including socialization and communication (3, 12). Early expressive and receptive language problems and behavioral problems may have long-term consequences (13). In particular, early receptive language problems are a significant risk factor for adult mental health (1).

However, the accuracy of detecting language problems in asymptomatic children in primary care is inadequate (14). Early recognition of adverse language development is challenging, given that normal development in young children is highly variable and all growth and development takes place in interaction with the environment (15–17). Differentiating between speech language delays and disorders is complicated, children with concerns about language development are a heterogeneous group with different individual and environmental characteristics. On the other hand, many children whose language development is delayed may catch up over the next few years and do not require interventions (18). Prevalence of language problems varies widely (2–25%) due to a lack of consistent definitions, the nature of the population, the diagnostic method that is utilized, and whether data were collected in a clinical sample or in the general population (19–21).

**Figure 1:**
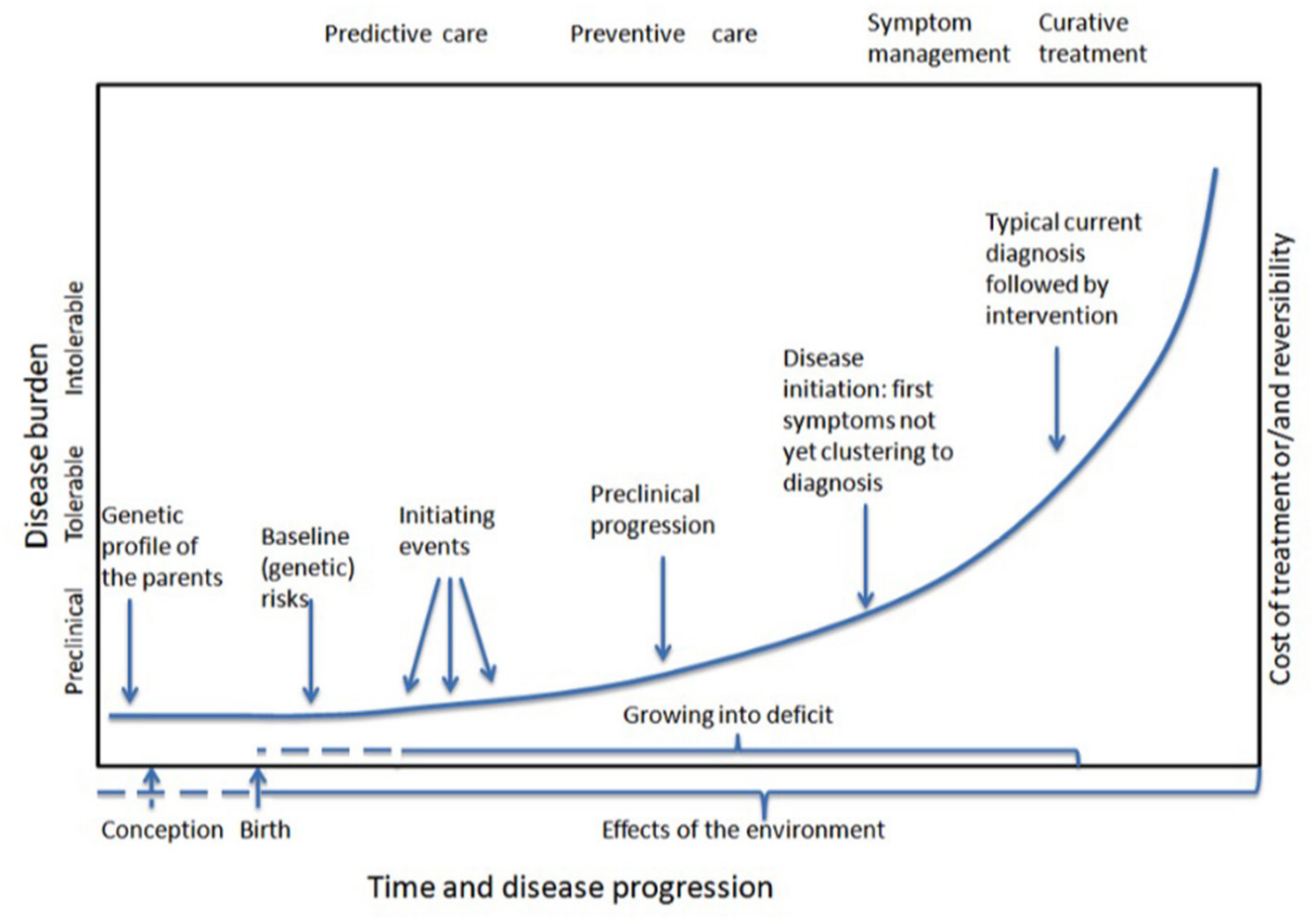
Modification of Syurina's adaptation of Snyderman's curve representing the timelines of “growing into deficit” and developing common complex diseases.

From a personalized health care “growing into deficit” model (Figure 1), prevention of language developmental problems requires a focus on concerns, emerging problems and symptoms at an early stage where signs and symptoms do not yet meet diagnostic criteria for a disorder (13, 22, 23). For early identification of language needs, it is important to understand the pervasive nature of language development (8, 13, 19). It is assumed that differences in young children's language development reflect differences in experience and in creating interactive routines, next to their biologically mediated genetic potential (24). If needed, early intervention has to be personalized; standard intervention programs have limited added value (25).

Previous research has shown that there is an association between language difficulties, behavioral difficulties, and social participation (26, 27). Language impairment in childhood may be related to problems with activities and social participation as defined by the International Classification of Functioning Disability and Health—Children and Youth (ICF-CY) (19, 28). Social participation is a broad concept including the objective state and the subjective experience of involvement in society. This concept has to be understood in the light of social roles (6). For young children, play is an important social activity.

Social competence development is linked with both language development and social participation. Social competence is affected when abilities or skills that are required to engage in socio-cognitive processes and to display social behaviors are limited (7). For example, not only expressive and receptive language, but also the ability to grasp non-linguistic signals is important for optimal social interaction and participation. Toddler's play has been associated with their language proficiency (4). However, this same study also showed that the child's functioning in play was better explained by their social competence than by their language skills. From a dynamic transactional developmental perspective (29), it is hypothesized that language development is mediated by social competence and social participation, and vice versa. Language development, social competence, and social participation are seen as dynamic skills simultaneously developing during the preschool period, suggesting a reciprocal model (30).

A community-based approach with a focus on personalized health care requires cooperation and communication within a public health framework (8, 22). According to a bio ecological model of development-in-context, it is important to obtain child context-specific information (20). Teachers, employees from childcare, kindergarten, preschool or primary school (hereafter: professional caregivers) as well as parents and Preventive Child Health Care (PCHC) professionals are important perceivers with expert knowledge on child development from different perspectives.

PCHC is synonymous with Pediatric Preventive Primary Care. All children in the area are regularly invited to visit the PCHC. The Dutch PCHC system includes preventive health care doctors and has a high level of population compliance. It is a public health endeavor to provide ongoing monitoring up to the age of 18 years (31, 32). This way, the early conditions that place children at risk for less than optimal development and successful social participation can be improved (33–36). To deal with emerging problems and symptoms at an early stage where signs and symptoms do not yet meet diagnostic criteria for a disorder, systematically exploring parental as well as other caregivers' concerns is a main component in PCHC for family-centered practice and personalized health care. Knowledge and understanding of the true epidemiology of genetic and environmental risk and protective factors and their early phenotypes can help in prevention of “growing into deficit” (23, 37).

In order to document children's development over time, monitoring development at multiple time points, across informants, instruments and contexts, is more valid and accurate than a single assessment (16, 38–41). For early identification of developmental problems, special attention should be given to the validity of instruments about the perceived impact of concerns as concurrent and long-term predictors, and outcome domains such as health, well-being and social participation (42). In a PCHC setting, monitoring instruments should: (1) easily obtain information in every day PCHC setting; (2) carry out dimensional assessment of symptoms and behavior; (3) measure the progress of development of young children and their possible determinants of influence; (4) identify general signals and symptoms indicating a possible disruption or imbalance of the educational/parent-child system, not yet related to a specific diagnosis; (5) support communication between PCHC, parents and professional caregivers about their perceptions on health and development; (6) connect to needs and demands of the child and the social system around the child; and (7) promote shared decision making (43, 44). Short instruments with a high negative predictive value are preferred; it ensures that most children who pass the developmental assessment are truly healthy. Follow-up consultations are no problem, these children can benefit from additional preventive monitoring (45).

Research has shown that parent-completed tools are highly accurate in detecting true problems, are relatively inexpensive, and promote a dialogue about concerns, needs and demands between parents and other caregivers (46–48). Therefore, incorporating tools utilizing a parent—and professional caregivers—report assessment like the Parents' Evaluation of Developmental Status (PEDS), child competence Visual Analog Scales (VAS) and the Strengths and Difficulties Questionnaire (SDQ) into a routine child monitoring toolkit could improve the rate of early identification of concerns about language development, social competence and social participation (43, 49). In this article, the concept social participation was operationalized using instruments to assess early emerging concerns about factors underlying preschool competence and social participation: a child's general competence at day care, kindergarten and preschool, the impact of distress and the total amount of concerns about child development and behavior.

This article investigates (1) the validity of the Parents' Evaluation of Developmental Status (PEDS) to assess language development concerns; (2) the cross-sectional association of language development concerns with social participation; (3) the longitudinal association of language development concerns with social participation, and (4) the possible mediating effect of social competence on the association between language development and social participation at the ages of 3 and 4 years.

## Methods

The present study was performed as part of the Monitoring Outcome Measurements of child development (MOM) study, a prospective observational study within PCHC practice. A community-based sample of 346 children was systematically assessed with a comprehensive PCHC “toolkit” of instruments using a multisource and cross-informant repeated measures design to identify developmental pathways impacting school readiness as an outcome of social participation. Children were aged 3 years at baseline and 4 years at follow up.

The Maastricht University Medical Center Medical Ethics Committee approved the MOM-study protocol under registration number MEC 09-04-018/P. Therefore, this study has been performed in accordance with the ethical standards laid down in the 1964 Declaration of Helsinki and its later amendments. All participating parents gave their informed consent prior to their inclusion in the study.

### Data Collection and Instruments

For this article, data of parents, professional caregivers and PCHC professionals of 341 children were analyzed. At baseline, children were aged 3 years and at follow-up ~4 years. To assess emerging problems, signs and symptoms, perceptions, demands and concerns about development and social participation, various short instruments like the Parents' Evaluation of Developmental Status (PEDS), the Strengths and Difficulties Questionnaire (SDQ) and child competence Visual Analog Scales (VAS) were included in this study. PCHC professionals provided information about, for example, background factors, family history, child health, and development and interventions.

#### Language Concerns

Parents as well as professional caregivers completed the PEDS, a 10-item standardized semi-structured questionnaire to elicit concerns regarding child development for children aged <8 years in the general population and clinical samples (40). Ten questions explore concerns in various domains: expressive and receptive language, fine motor, gross motor, behavior, socialization, self-care and learning. The PEDS-question could be answered on a trichotomous scale: “no,” “a little,” “yes.” Subsequently, an open-ended field provides more information. The PEDS is validated for clinical samples and general population samples aged between 0 and 8 years, and is available in multiple languages. In recent validation studies from the USA for the accuracy of parental concerns in detecting children at high and/or moderate developmental risk, the PEDS has a sensitivity of 91–97% and specificity of 73–86% (50). The PEDS is less time-consuming than other instruments, emphasis is on parental and other professional caregivers' opinions, and has reasonable test characteristics for developmental screening in primary care settings (51). Furthermore, the PEDS has shown to be reliable, valid and useful as brief monitoring tools in daily Dutch PCHC practice (43, 51). This suggests that the PEDS is an accurate tool for use as an initial screening and monitoring tool in Dutch PCHC, where professionals have to deal with the time constraints of daily practice.

For the current paper, dichotomous “parental concerns” and “professional caregiver concerns” variables about expressive and/or receptive language (any concern yes/no) were constructed for use in the analyses, if any of the parents or professional caregivers scored “yes” or “a little,” the answer was recoded as “yes.”

#### Child Competence

To address the issue of the child's functional adaptation, professional caregivers were asked to indicate on 2 VAS, the degree of the child's general competence and the child's social competence (0 = not competent, 100 = very competent).

#### Participation

The child's general competence as described above is one of the instruments to assess the broad construct of participation. Other instruments are SDQ total score and SDQ impact.

The Dutch version of the SDQ was completed by parents as well as by professional caregivers to assess the child's behavior (46, 52–54). The SDQ is a brief behavioral screening questionnaire for children aged 3–16 years. It also includes items that identify the impact of the behavioral problems of the child. The SDQ is considered valid and reliable as a research instrument in community samples (49). For this article, the “SDQ total sum score” and the “SDQ impact of distress score” of both parents and professional caregivers were used. If any of the parents or professional caregivers scored “yes” on the impact probe question, the dichotomous overall distress variable was set at “yes.”

#### Van Wiechen Developmental Test

In addition to the validation of the overall PEDS, validity of the PEDS language items was assessed, using the Van Wiechen developmental test as reference standard (43, 55–57). This Dutch instrument is a modification of the Gesell test and is routinely used by all PCHC Centers in the Netherlands and Belgium to monitor the development of all children from birth to the age of 4 years. It consists of a set of 57 developmental indicators to assess motor behavior, speech, communication, and social skills based on physicians' observations and interviewing the parents. A total of 23 indicators cover language development and communication and are called language milestones. All PCHC professionals are trained to asses and register mile stones in the PCHC system according to a uniform protocol. For this paper, the Van Wiechen communication and language items were used. In a large community-based sample of Dutch children, test characteristics of the Van Wiechen language items for the age group 36–48 months showed an Area Under the Curve (AUC) of 0.83%, with an average sensitivity of 66.1%, specificity of 87.5%, positive predictive value (PPV) of 29.2%, and a negative predictive value (NPV) of 98.8% (58).

In a study in Australia, agreement between ratings of parental PEDS language concerns and clinical assessment was high (86–90%); agreement between teacher PEDS language concerns and clinical assessment was lower and more varied (63–80%) (59). In this study, parental and professional caregiver PEDS language concerns were combined to provide complementary information and capture all possible language concerns of a specific child. Subsequently this combined concerns variable was validated; reference standard was the Van Wiechen developmental test, communication and language items (see above) (60, 61). For this study, the PCHC professionals were asked to judge the Van Wiechen language and communication items as “sufficient” or “not sufficient,” at the age of 3 years and a year later.

#### Other Variables

As an indicator of socioeconomic status, the level of maternal and paternal education was assessed across three categories: low (primary education, junior vocational education), middle (general secondary education, senior vocational education) and high (preparatory university education and university education). The parent with the highest level of education determined parental educational level.

### Statistical Analyses

All analyses were performed using Stata Statistical Software, version 15 (62). First, to assess the validity of the PEDS language items, positive predictive value (PPV) and negative predictive value (NPV) were obtained at the age of 3 years and at the age of 4 years. PPV and NPV were then assessed as measures of predictive validity of language concerns at the age of 3 years, using the Van Wiechen developmental test at age 4 years as reference standard. Second, logistic and linear regression analyses were performed. In the cross-sectional analyses at both ages 3 and 4 years and in the longitudinal analyses, the independent variable (X) was concerns about language development. The dichotomous dependent variable (Y) to index social participation, used in the logistic regression analyses, was SDQ impact of distress score. Continuous dependent variables (Y) to index social participation, used in the linear regression analyses, were: SDQ total score, the child's general competence VAS and the child's social competence VAS. Analyses were adjusted a priori for age, sex, and parental educational status.

**Figure 2:**
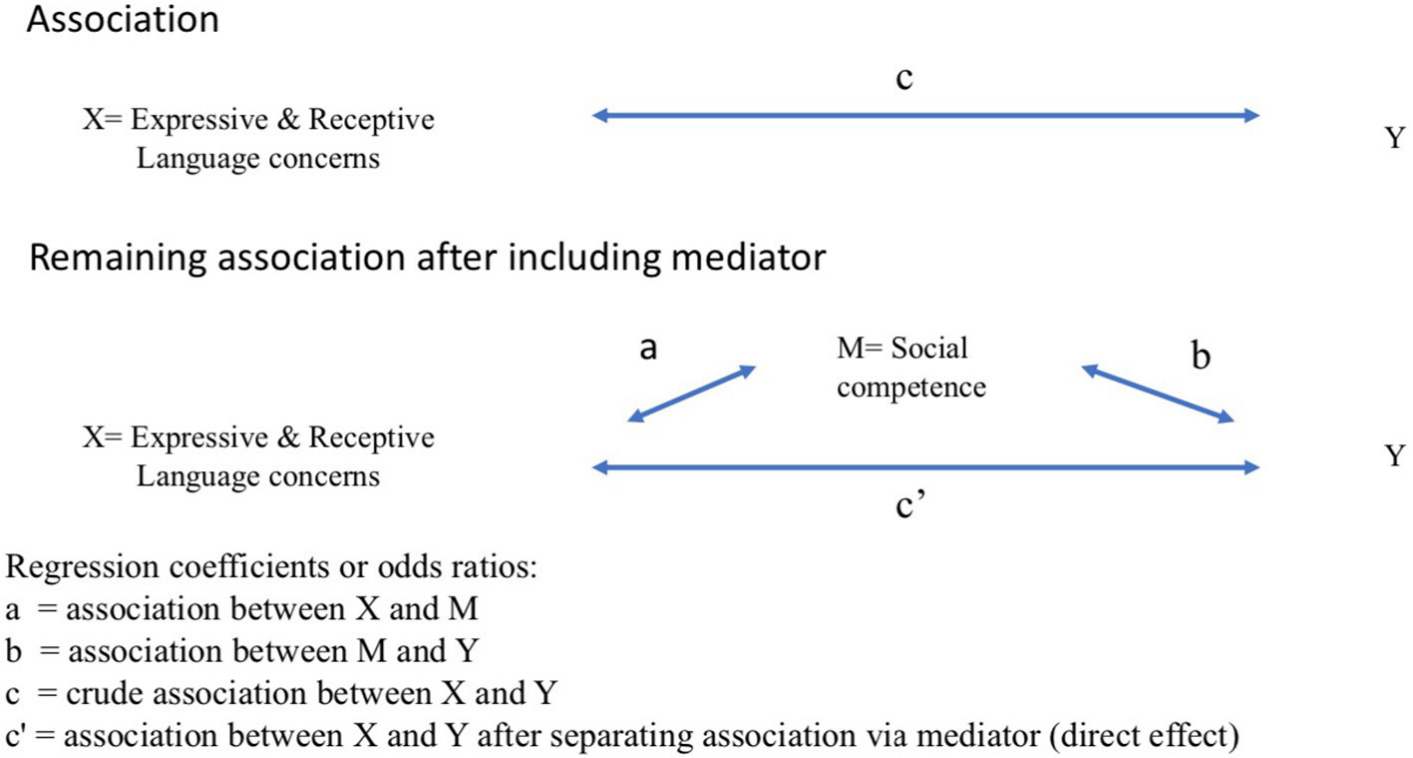
Theoretical figure to explain analysis of mediation.

Finally, to analyze the fourth research question, the child's social competence was included as a mediator (M) in the association between independent (X) and dependent (Y) variable. Mediation was assessed by analyzing a regression model with and without the mediator. The question was whether the association between X and Y after including the mediator is zero or substantially smaller than the direct association between X and Y. This is visualized in Figure 2. The arrows a, b, c and c' present regression coefficients or odds ratios: a represents the association between independent variable (X) and mediator (M); b represents the association between the mediator (M) and the dependent variable (Y); c represents the crude association between independent variable (X) and dependent variable (Y); and c' represents the association between X and Y after including the mediator (M) in the regression model. When the hypothesis that there can be mediation is plausible and c shows an association while c' is smaller or close to zero, there is evidence for partial or full mediation, respectively.

## Results

Parents of 346 children agreed to participate in the MOM study. At baseline, parents of 341 children and professional caregivers of 301 children completed the questionnaires. The mean age of the children was 3.0 years (SD 0.2, Table 1). For 296 of these children (86%), information from both informants was available. In the follow up, at the age of ~4 years (mean age 3.8; SD 0.2, Table 1), information of both informants was available for 236 children (68%). For 32 children (9%) there was no information available from parents or professional caregivers because they did not return the questionnaire. At baseline, the total sample of children consisted of 166 boys (48%) and 180 girls (52%). Of the participating children, 60% (*n* = 207) were resident in the municipality of Maastricht, while 40% (*n* = 139) lived in the surrounding areas. At baseline, parents and/or professional caregivers of 108 (32%) of 334 children had concerns about expressive and/or receptive language development (12 missings on the PEDS). In the follow up, at the age of 4 years, the total number of children with concerns about language development was 81 (26%) of 313 children (Table 1).

**Table 1:** Descriptive statistics at baseline (T1) and follow-up (T2).

**Variable**	***N***		**Mean (S.D.)**		**Range**	
	**T1**	**T2**	**T1**	**T2**	**T1**	**T2**
Age in years	346	293	3.0 (0.2)	3.8 (0.2)	1.8–3.5	3.5–4.8
Child general competence VAS^a^	290	251	63.7 (19.7)	69.7 (16.3)	4–100	10–99
Child social competence VAS^a^	297	254	62.9 (23.4)	68.9 (20.1)	3–100	9–99
SDQ (parents)	338	293	6.8 (4.9)	6.1 (4.2)	0–28	0–27
SDQ (prof.^b^)	294	256	6.1 (5.0)	5.0 (5.0)	0–27	0–29
			**Normal**		**Atypical**	
PEDS concerns about language^c^	334	313	226 (68%)	232 (74%)	108 (32%)	81 (26%)
Van Wiechen developmental test ^d^	331	319	304 (92%)	298 (93%)	27 (8%)	21 (7%)
SDQ impact (parents)	340	292	307 (90%)	271 (93%)	33 (10%)	21 (7%)
SDQ impact (prof.^b^)	292	254	248 (85%)	223 (88%)	44 (15%)	31 (12%)

a *A higher Visual Analog Scale (VAS) score means professional caregiver judges child competence more positive*.

b *Professional caregivers*.

c *Parental and/or professional caregiver's concerns about expressive and/or receptive language development*.

d *Speech, language and communication items*.

In order to test representativeness, 40% of non-responders were randomly sampled to manually collect data on parental education from the medical files. The distribution in non-responders was minimally different from distribution in responders (responders 63, 27, and 10% and non-responders 55, 33, and 12% having high, intermediate and low parental education, respectively).

### Validity of the PEDS Language Items

The prevalence of PCHC language concerns was 8% at the age of 3 and 7% one year later (Table 2). At the age of 3 years, PEDS language concerns had a PPV of 23% and NPV of 99%. At the age of 4, the PPV and NPV of PEDS language concerns were 19 and 97% respectively (Table 2). Table 3 shows the stability of language developmental concerns at the age of 3 and 4 years. The predictive validity of the PEDS at the age of 3 years was: PPV of 14% and NPV of 97% (Table 4).

**Table 2:** Prevalence, Positive, and Negative Predictive Value of PEDS concerns about language development at the age of 3 and 4 years.

**Expressive language and/or receptive language**	**T1 reference standard**^**b**^		
**T1PEDS concerns**^**a**^	**Yes**	**No**	**Total**
Yes	24	82	106
No	3	222	225
Total	27	304	331
**Expressive language and/or receptive language**	**T2 reference standard**^**b**^		
**T2 PEDS concerns**^**a**^	**Yes**	**No**	**Total**
Yes	15	65	80
No	6	225	231
Total	21	290	311

*T1 Prevalence 8.2%; Specificity 73%; Sensitivity 88.9%; PPV 22.6%; NPV 98.7%*.

*T2 Prevalence 6.8%; Specificity 77.6%; Sensitivity 71.4%; PPV 18.8%; NPV 97.4%*.

a *Parental and/or professional caregiver's concerns about language development*.

b *Van Wiechen developmental test, speech, language and communication items*.

**Table 3:** Stability of PEDS concerns about language development at the age of 3 and 4 years.

**Expressive and/or receptive language**	**T2 PEDS concerns**^**a**^		
**T1 PEDS concerns**^**a**^	**Yes**	**No**	**Total**
Yes	55 (18%)	45 (14%)	100 (32%)
No	26 (8%)	186 (60%)	212 (68%)
Total	81 (26%)	231 (74%)	312(100%)

*Prevalence 26.0%; Specificity 80.5%; Sensitivity 68.0%; PPV 55.0%; NPV 87.7%*.

a *Parental and/or professional caregiver's concerns about language development*.

**Table 4:** The predictive validity of PEDS concerns about language development at the age of 3 years.

**Expressive and/or receptive language**	**T2 reference standard**^**b**^		
**T1 PEDS concerns**^**a**^	**Yes**	**No**	**Total**
Yes	14	89	103
No	7	209	216
Total	21	298	319

*Prevalence 6.6%; Specificity 70.1%; Sensitivity 66.7%; PPV 13.6%; NPV 96.8%*.

a *Parental and/or professional caregiver's concerns about language development*.

b *Van Wiechen developmental test, speech, language, and communication items*.

### Association Between Language Development Concerns and Preschool Social Participation

When assessing parental SDQ impact at the age of 3 years, children with receptive language concerns had an OR of 7.3 and children with expressive language concerns had an OR of 2.4. However, confidence intervals were overlapping (Table 5).

**Table 5:** Logistic regression analysis (significant interaction with one or both risk factors): association between PEDS concerns about expressive and receptive language development and SDQ impact according to parents and professional caregivers at the age of 3 and 4 years; odds ratios (OR) and 95% confidence intervals (CI).

	**SDQ impact (Parents)**		**SDQ impact (prof**.^**b**^**)**	
	**T1**	**T2**	**T1**	**T2**
	**OR (95% CI)**	**OR (95% CI)**	**OR (95% CI)**	**OR (95% CI)**
PEDS concerns^a^ Expressive language	2.4 (1.2; 5.1)^*^	2.5 (1.0; 6.3)^*^	3.0 (1.5; 5.8)^**^	2.3 (1.1; 5.0)^**^
PEDS concerns^a^ Receptive language	7.3 (3.1; 17.3)^†^	5.3(1.7; 16.3)^**^	10.5 (4.6; 24.1)^†^	2.6 (0.8; 8.6)

* *p < 0.05*;

** *p < 0.01*;

† *p < 0.001*;

*95% CI, 95% confidence interval; OR, odds ratio (obtained from logistic regression)*.

a *Parental and/or professional caregiver's concerns about language development*.

b *Professional caregivers*.

According to professional caregivers, the association between receptive language concerns and outcomes was stronger than the association between expressive language and outcomes (e.g., general competence VAS: B = −21.3, *p* < 0.001; Table 6). In addition, both professional caregivers and parents reported more behavioral problems when there were receptive language concerns (SDQ total score B = 4.5, *p* < 0.001; B = 4.5, *p* < 0.001, respectively). A year later, the association between language concerns, competence and behavior was less strong but still significant, except for PEDS expressive language concerns and the parental perception of child behavior (Table 6).

**Table 6:** Linear regression analysis: association between PEDS concerns about expressive and receptive language development and child general competence, child social competence, and total score SDQ according to parents and professional caregivers at the age of 3 and 4 years; b- coefficient (B) and 95% confidence intervals (CI).

	**Child general competence VAS**^**b**^ **(prof**.^**c**^**)**		**Child social competence VAS**^**b**^ **(prof**.^**c**^**)**		**Total score SDQ (Parents)**		**Total score SDQ (prof**.^**c**^**)**	
	**T1**	**T2**	**T1**	**T2**	**T1**	**T2**	**T1**	**T2**
	**B (95% CI)**	**B (95% CI)**	**B (95% CI)**	**B (95% CI)**	**B (95% CI)**	**B (95% CI)**	**B (95% CI)**	**B (95% CI)**
PEDS concerns^a^ Expressive language	−11.4 (−16.4 −6.8)^†^	−10.7 (−15.1; −6.2)^†^	−12.1(−17.9; −6.4)^†^	−10.3 (−15.9; −4.7)^†^	2.6 (1.5; 3.7)^†^	1.1 (−0.0; 2.3)	2.8 (1.5; 4.0)^†^	2.2 (0.8; 3.6)^**^
PEDS concerns^a^ Receptive language	−21.3 (−28.7; −13.9)^†^	−13.3 (−21.7; −4.9)^**^	−19.1 (−27.8; −10.4)^†^	−16.1 (−26.5; −5.7)^**^	4.5 (2.7; 6.3)^†^	4.2 (2.3; 6.0)^†^	4.5 (2.7; 6.3)^†^	3.2 (0.8; 5.7)^**^

_*_p < 0.05;

** *p < 0.01*;

† *p < 0.001*;

*95% CI, 95% confidence interval; b–coefficient (B) (obtained from linear regression)*.

a *Parental and/or professional caregiver's concerns about language development*.

b *A higher VAS score means professional caregiver judges child competence more positive*.

c *Professional caregivers*.

### Mediating Effect of Social Competence

At baseline and at follow up a year later, there was a significant association between social competence and social participation, but also a direct association between concerns about expressive and/or receptive language development and social competence. For example, according to the professional caregiver, at age 3 years there was a significant association between language concerns and social participation (SDQ impact B = 4.3, *p* < 0.001; SDQ total score B = 3.0, *p* < 0.001) and a significant association between social competence and social participation (SDQ impact B = 0.9, *p* < 0.001; SDQ total score B = −0.1, *p* < 0.001) (Tables 7, 8). There was a mediating effect of social competence: after inclusion of social competence in the regression model, the remaining association between language concerns and social participation was less strong (SDQ impact B = 2.8, *p* = 0.014; SDQ total score B = 1.2, *p* = 0.016). A year later, at the age of 4 years, the mediating effect of social competence was even stronger with non-significant regression coefficients (B = 1.1, *p* = 0.884 and B = 0.8, *p* = 0.198, respectively). When tested separately, the mediating effect was found both for expressive and receptive language concerns (data not shown). Mediating effects of social competence were also found between language concerns and general competence (data not shown).

**Table 7:** Mediating effect of social competence on the association of PEDS concerns about language development with SDQ impact score at T1 and T2.

		**Path**	**Odd ratios**			
			**T1**		**T2**	
			**Parent**	**Prof.**^***b***^	**Parent**	**Prof.**^***b***^
PEDS concerns^a^ language (X)	Social competence (M)	A	−13,5^*†*^^*c*^	−13,5^*†*^^*c*^	−10.8^*†*^^*c*^	−10.8^*†*^^*c*^
Social competence (M)	SDQ Impact score (Y)	B	1.0^**^	0.9^†^	1.0^†^	0.9^†^
PEDS concerns^a^ language (X)	SDQ Impact score (Y)	C	4.2^†^	4.3^†^	2.8^*^	2.4^*^
PEDS concerns^a^ language (X)	SDQ Impact score (Y)	C'	3.0^**^	2.8^*^	0.9	1.1

* *p < 0.05*;

** *p < 0.01*;

† *p < 0.001*.

a *Parental and/or professional caregiver's concerns about expressive and/or receptive language development*.

b *Professional caregivers*.

c *B regression coefficients*.

*Odd ratios unless otherwise indicated*.

*A = Association between X and M*.

*B = Association between M and Y*.

*C = Crude association between X and Y*.

*C' = Association between X and Y after separating association via mediator (direct effect)*.

**Table 8:** Mediating effect of social competence on the association of PEDS concerns about language development with SDQ total score at T1 and T2.

		**Path**	**B regression coefficient**			
			**T1**		**T2**	
			**Parent**	**Prof.**^***b***^	**Parent**	**Prof.**^***b***^
PEDS concerns^a^ language (X)	Social competence (M)	A	−13,5^†^	−13,5^†^	−10.8^†^	−10.8^†^
Social competence (M)	SDQ total score (Y)	B	−0.0^†^	−0.1^†^	−0.1^†^	−0.1^†^
PEDS concerns^a^ language (X)	SDQ total score (Y)	C	3.2^†^	3.0^†^	1.5^**^	2.4 ^**^
PEDS concerns^a^ language (X)	SDQ total score (Y)	C'	2.1^†^	1.2^*^	0.1	0.8

* *p < 0.05*;

** *p < 0.01*;

† *p < 0.001*.

a *Parental and/or professional caregiver's concerns about expressive and/or receptive language development*.

b *Professional caregivers*.

*B regression coefficients*.

*A = Association between X and M*.

*B = Association between M and Y*.

*C = Crude association between X and Y*.

*C' = Association between X and Y after separating association via mediator (direct effect)*.

## Discussion

The results in this paper suggest concurrent and predictive validity of the PEDS to assess parental and professional caregivers' language development concerns, as well as the mediating effect of professional caregivers' perception of the child's social competence in the association between these concerns and social participation at the age of 3 years as well as the age of 4 years.

### Validity of Preschool Language Development Concerns

Prevalence of language delay (7–8%) in the present study is within the international reported range of prevalence of atypical language delay (7–15%) (20, 21, 38). In addition, the association between parental and professional caregiver concerns on the one hand, and not meeting the expected milestones for language on the other, was statistically significant. These results are in line with other studies where parental concerns were consistently associated with preschool language development (40, 58, 63). Moreover, this confirms the value of including parents' and professional caregivers' expert knowledge in the assessment and clinical decision-making process for personalized support (28, 59). The high NPV of parental and professional caregiver language concerns validate a strategy of exclusion of children without concerns from extra monitoring. The PEDS language screening items appear to be very good in identifying children who do not have any language delay. Current assessment tools are still not sufficiently specific to discriminate between delayed language that will resolve naturally and delayed speech or language that will develop into persistent problems. The relatively low PPV in the present study implies a high percentage of false positives. Earlier research has shown that children with false positive screening results differ from children with true negative scores. These children had more risk factors and their performance on diagnostic measures was less (45). As confirmed by other population studies (17, 18, 26, 64), this article showed that while some preschool children grow out of language problems, others may develop them (Table 3). From a classical screening point of view, children crossing back and forth over the threshold would impact sensitivity and specificity. However, PCHC repeated monitoring concerns of language development and if necessary, extra follow up can make a distinction between children “growing into or out of deficit.”

So, PCHC monitors the true positives as well as the false positives and refers when needed, even if the child did not score on the reference standard. Language tests may not capture important aspects of everyday communication. In addition, a language problem may not always look like a language problem: underlying comprehension impairment can present as poor academic attainment, impaired social interaction, or behavioral difficulties (12, 65). Furthermore, due to the variation in the cut-off points of different “reference standard” measures in research, interpretation of parents' and professional caregivers' information is complicated. In addition, there is no agreement on different definitions of language disorders and what proportion of the population should be considered cases that need intervention (65).

### Prediction of Preschool Social Participation: Mind the Communication

Parental and professional caregiver concerns were associated with altered social participation at home as well as in preschool. This association was seen both in cross-sectional and in longitudinal analyses.

Language concerns seem to be predictive for altered social participation as early as in preschool. Earlier research showed that especially children who experienced language impairment that persisted into the school years are at risk for adult mental health problems and substandard social participation (66). The strongest association was seen between receptive language delay concerns and behavioral problems. Odds ratio confidence intervals of children with receptive and expressive language concerns were overlapping, thus were not statistically significant, except for the association with parental SDQ total score at the age of 4 years (Table 5). From PCHC practice it is recognizable that receptive and expressive language development are closely linked, with more problems in social participation because of language comprehension problems. This confirms the observation that needs of children with receptive language problems are complex and call for extra monitoring of the child's developmental pathway (1). Listening to parental and professional caregivers' concerns with avoidance of diagnostic labels is an important aspect of PCHC clinical judgement and pre-screening. It may identify other developmental problems without potential stigmatization (23, 37). Avoidance of diagnostic labels is not the same as denying any role of biological risk factors in causing health problems; children vary in their biological as well in their social backgrounds and life events (65).

### Mediating Effect of Social Competence

Language delay in itself may not be a risk factor for later behavioral and emotional disturbances (67). The present results showed that concerns about language development may reflect the effect of other developmental problems (68). There was a mediating effect of child social competence on the association between receptive and expressive language concerns and social participation at the age of 3 and 4. While at age 3 years social competence was a partial mediator, at age 4 it was a full mediator. So, at age 4, social competence seems to play a more important role in the association between concerns and participation. After inclusion of social competence score, language concerns seem to lose their predictive value but these factors might be related to each other.

The expansion of this mediating effect between ages 3 and 4 years emphasizes once again that all children with language concerns can benefit from additional monitoring to prevent “growing into deficit,” especially concerning interpersonal relationships. There is a role for enhanced monitoring in which the primary care professional responds to parental concerns about language development and social skills (3, 6, 23). The group of children with symptoms of mental problems may be twice as large as the group of children meeting formal diagnostic criteria for a mental disorder (69–77). Inefficiency can arise if educational and medical support is restricted to those who meet arbitrary cut-offs as a result of discrepancy in criteria used for diagnostic labels (65). Therefore, a PCHC “toolkit” with short instruments for regular short parental and professional caregivers' reports can serve as a first step in PCHC monitoring procedures to select children who require further support in the form of a “watch and wait” strategy, assessment of other developmental domains, or referral to a specialist. Professional support can then be tailored to the needs, conform the child's development.

PCHC professionals have to deal with emerging problems and symptoms at a stage where signs and symptoms do not yet meet diagnostic criteria, but already give rise to early impairment and distress for both the children and their context, at home as well as in preschool. Both parental and professional caregiver concerns are relevant for early detection of problems, because they both know the child and their perception is from a different perspective (41). The PEDS: (1) facilitates monitoring of parental and professional caregivers concerns; (2) identify general signals and symptoms not yet related to a specific diagnosis; (3) support communication between PCHC, parents and professional caregivers about their perceptions on health and development; and (4) promote shared decision making (23, 44).

## Methodological Issues

Strength of the study is that a community sample of preschool children was systematically assessed using a comprehensive PCHC “toolkit” of instruments designed for the purpose of monitoring in a public health setting. The study was integrated in real life practice. No children were excluded for not meeting inclusion criteria. In addition, the child's development and participation was evaluated across different settings with cross-sectional and longitudinal information from different instruments and multiple informants. With emphasis on their perception, information was obtained through hetero-anamnesis of parents and professional caregivers (78). PCHC professionals provided data as well; with exception of the Van Wiechen developmental test, these data were not used in the present paper. Furthermore, the Van Wiechen language milestones were collected in a uniform manner by trained professionals.

The present paper has some limitations. First, response rates were difficult to establish. In the MOM region over the study period, 1,692 children were born and, therefore, were within the caseload of the PCHC professionals participating in the MOM study. However, not all PCHC professionals participated in MOM. Consequently, parents of non-participating PCHC doctors were asked to participate by another PCHC doctor (BD), who did not know these families. During the baseline inclusion, the number of participating PCHC professionals increased. Response from one PCHC doctor who participated from the beginning (BD) was 70%. Because response in participating doctors was relatively high and because distribution in socio-economic status was comparable, results presented in this article can be considered approximately representative for the general population. If the PEDS and different VAS are implemented in general PCHC practice, a response rate higher than 70% is expected, because a possible barrier for parents to participate in MOM was the number of questions added for research purposes (e.g., additional instruments for the purpose of validation of VAS). Usually, short form questionnaires collected in PCHC have response rates between 80 and 90% (79).

Second, the MOM data are limited to the city of Maastricht and surrounding areas. This part of the Netherlands is quite similar to the rest of the country. However, there are some differences. In Maastricht, the proportion of non-European inhabitants (about 10%) is less than in the larger cities in the north west of the country (about 30%) (80). In addition, the proportion of highly educated parents participating the MOM is quite large (63%). For this reason, the MOM study findings may not necessarily be valid for large cities with ethnically mixed populations and areas with a larger proportion of low educated parents.

Third, assessment tools were general in nature and did not reveal specific information to assist with remediation of deficits. The measures find general delay and it is not necessarily clear that the delays are clinically significant. PCHC professionals must remain aware they have to deal with emerging problems and symptoms at a stage where signs and symptoms do not yet meet diagnostic criteria. Although each type of parental concern can be associated with validated tests on the same developmental domain, studies about the validity of the PEDS showed that parents often have concerns in seemingly unrelated domains [i.e., parents often reflect on not just the apparent problem but also its impact on other aspects of development (40)]. Revelation of parental and professional caregivers' concerns are a first step in PCHC monitoring procedures to select children who require further assessment of other developmental domains, or referral to a specialist.

Fourth, social competence was included as a mediator and this was based on judgement of the professional caregiver. However, if a similar instrument was available from the parents, we would expect to find similar mediating effects.

Finally, because of the small sample size, analyses were not adjusted for risk factors (e.g., family history of language or literacy problems, health or developmental problems).

## Conclusions

The individual's social and educational environment, including interpersonal relationships, is hypothesized to be instrumental for PCHC professionals wishing to provide personalized preventive public health care for successful participation for all children (31). In order to identify emerging problems at an early stage where signs and symptoms do not yet meet diagnostic criteria for a disorder, short but valid PCHC monitoring tools like the PEDS and different VAS are required (43). Within this PCHC “toolkit,” parental and professional caregivers' perception and concerns about language development take an important position. Language development can be seen as the outcome of the mental processes set in motion when the child meets the social and linguistic world (24, 81). The analyses presented here uncovered significant associations between parental and professional caregiver concerns about language development, the child's social competence and the level of preschool social participation. Therefore, pediatric primary care professionals may productively use parental and professional caregiver perceptions concerning preschool language development in clinical practice. Equally important is the perception of a child's social competence. In children not meeting the expected milestones for language development, a comprehensive developmental evaluation and additional monitoring of child development may be required, particularly concerning interpersonal relationships. Consequently, personalized health care requires cooperation within the public health frame. Monitoring of language and social competence development in preschool children can profit from continued communication between parents, professional caregivers and preventive child health care.

## Data Availability Statement

The datasets for this article are not publicly available to conform with the General Data Protection Regulation, a European regulation regarding the processing of personal data. Requests to access the datasets should be directed to Bernice Doove, MD, bernice.doove@ggdzl.nl.

## Ethics Statement

The Maastricht University Medical Center Medical Ethics Committee approved the MOM-study under registration number MEC 09-04-018/P. Therefore, this study has been performed in accordance with the ethical standards laid down in the 1964 Declaration of Helsinki and its later amendments.

## Author's Note

Underlying research materials related to this paper are available on request by the corresponding author.

## Author Contributions

BD drafted the initial manuscript. FF, JO, and MD reviewed and revised the manuscript. All authors approved the final manuscript as submitted, agreed to be accountable for all aspects of the work, and conceptualized and designed the study.

## Conflict of Interest

The authors declare that the research was conducted in the absence of any commercial or financial relationships that could be construed as a potential conflict of interest.

## Abbreviations

PCHC: Preventive Child Health Care
PEDS: Parents' Evaluation of Developmental Status
SDQ: Strengths and Difficulties Questionnaire
Stata: Statistical Software Package
VAS: visual analog scale.
